# Process evaluation of postpartum contraceptive service delivery in Ayder Comprehensive Specialized Hospital Mekelle, Tigray, Ethiopia in 2020

**DOI:** 10.1186/s12913-023-09467-8

**Published:** 2023-07-25

**Authors:** Kahsay Negash Hagos, Abreha Addis Gesese

**Affiliations:** 1 Department of Monitoring and Evaluation, Mekele University, Northern, Ethiopia; 2 Department of Clinical Nursing, Gambella Teachers Education and Health Science College, Gambella, Ethiopia

**Keywords:** Process evaluation, Quality service, Postpartum contraceptive, Women, ACSH, Tigray

## Abstract

**Background:**

Postpartum contraceptives during the first year after delivery is a key service for women to prevent unintended pregnancy and reduce the risk of maternal and child mortality by ensuring safe birth intervals. This process evaluation aimed to assess the availability, compliance, and accommodation of Post-Partum Contraceptives (PPC) and the experience of women’s contraception in the first 12 months postpartum in Ayder Comprehensive Specialized Hospital (ACSH).

**Methods:**

A case study evaluation design with a mixed method was employed from February 16/2020 to Mar 30, 2020. Direct observations, 12-month document reviews, and key informant interviews were conducted. The quantitative data were entered into Epi-Data version 3.1 and exported to SPSS version 21 for analysis. In the multivariate logistic regression analysis, variables with < 0.05 *p*-values and Adjusted Odds Ratio (AOR) with 95% Confidence Interval (CI) were used to declare the association. The qualitative data were transcribed, translated, coded, and analyzed using thematic analysis. The overall process of program implementation was measured based on pre-determined judgmental criteria.

**Result:**

From the total of charts reviewed 302 only 188 (62.3%) postpartum mothers used any method within 12 months of the postpartum period out of which only 27.1% mothers used of long-acting reversible contraceptive (LARC). The overall evaluation of postpartum contraceptives was 84.1% (V/good). Notable gaps observed in this study were poor provision of information in relation to methods given, poor technical performance in following the aseptic procedure, poor utilization of postpartum family planning guidelines and clinical checklists for counseling, and poor use of information education materials compared to the national standards. Residence, number of stillbirths or neonatal loss, counseling status of family planning during ANC visits, and maternal counseling status of family planning during postnatal care visits were factors associated with PPC.

**Conclusion:**

The overall postpartum contraceptive service delivery in ACSH was V/good compared to the national family planning guideline standards. With the notable gaps identified, specific recommendations were suggested to different responsible bodies.

## Background

Postpartum contraception is used to prevent unintended and closely spaced pregnancies in the first 12 months after child birth. It is a critical time to address high unmet family planning need and to reduce the risks of closely spaced pregnancies [1]. The World Health Organization (WHO) recommends that after “a woman gave birth” she should avoid pregnancy for at least two years prior to the next pregnancy to ease the maternal, pre-natal and infant outcome related risks [2]. Practical tools are included in the resource for integrating postpartum family planning at points when women have frequent health system contact, including during antenatal care, labor and delivery, postnatal care, immunization, and child health care [3].

A comprehensive Postpartum Family Planning(PPFP) intervention entails continuity of care for the woman and her baby, reduce maternal, infant, and childhood deaths [1, 4], reductions high-risk pregnancies, as well as in improvements in schooling and economic outcomes, especially for girls and women [5]. Pregnancy within 12 months after childbirth is at increasing risk of preterm- birth and neonatal death [6].

Current use of modern contraceptives prevents an estimated 307 million unintended pregnancies annually among adolescent girls and women in developing regions. Meeting the unmet need for modern contraception among adolescents aged 15–19 would reduce unintended pregnancies in this age group by 6 million annually, thereby averting 2.1 million unintended births, 3.2 million abortions, and 5,600 maternal deaths. The demand for family planning that is satisfied with modern contraception remains below 50% in many low-income countries [7]. Over the past five decades, the use of FP method has steadily increased in SSA with percentage of married women using modern contraceptives ranging between < 20% and 69%. Unmet need for FP is unacceptably high [8].

In Ethiopia, around 18% of women used modern contraception within the first 12 months after child birth. The pregnancy related mortality ratio (MMR) was 412 maternal deaths per 100,000 live births (LBs), and the infant mortality and under-five child mortality rates were 48 and 67 deaths per 1,000 LBs, respectively [9]. Postpartum contraceptive use helps women to realize their desire of birth spacing and to avoid unplanned pregnancies [10]. Unlikely, a study in Hossana town showed relatively higher postpartum contraceptive [11].

In Tigray region, the overall contraceptive prevalence rate among all women was 623 (35.6%) while 543 (41.0%) among married women. Depo-Provera was the most common type of contraceptive used as mentioned by 402 (64.5%) of the women [12], 33.8% postpartum mothers used modern contraceptives in rural setting of Tigray, in which Depot medroxyprogesterone acetate and implant were the most prevalent methods used [13]. A recent evidence from Adigrat indicated that the post-partum of long acting reversible contraceptive (LARC) service utilization was as low as 5.8% (comprised of 5.3% Implants and 0.5% Intrauterine device (IUD)), while the short acting family planning service coverage was as high as 94.2% [6].

Despite the increasing general family planning utilization rate over time in ACSH, the evaluation assessment indicated that postpartum contraceptive service utilization among mothers within the first twelve months was low after delivery. The HMIS report of 2018/19 fiscal year on PPFP, only 1179(25.24%) mothers used PPC out of 4670 delivered women in ACSH. Similarly, during the first half of 2019 fiscal year, 614(25%) received PPC out of 2466 women delivered in ACSH. Most women preferred the short acting methods. In an effort to achieve a better future for all, SDG 3 has targeted to reduce maternal mortality ratio to less than 70 per 100,000 live births by 2030 and plans to ensure universal access to sexual and reproductive health-care services; including for family planning [14]. All services that clients receive must be consistently high quality, monitored and evaluated. Meanwhile, the finding will help to improve the service delivery by averting program implementation gaps. Therefore, this process evaluation was aimed to assess the postpartum contraceptive service delivery in Ayder Comprehensive Specialized Hospital (ACSH) in three dimensions such as the availability; compliance; and accommodation within 12 months.

## Evaluation methods

### Study design, area and period

A case study evaluation design with a mixed method was employed from February 16/2020 to Mar 30, 2020 in ACSH of Mekelle city. A case study is a research approach that is used to generate an in-depth, multi-faceted understanding of a complex issue in its real-life context. It needs to explore an event or phenomenon in depth and in its natural context [15]. Mekelle city of Tigray state is located at about 783 kms North of Addis Ababa. It has seven sub-cities with an estimated population of 332,013 with projected according to low, medium and high growth rate scenarios for the year of 2015 assuming a medium growth rate of 2.7%, of which 51.4% were female. The majority of the people were Tigrians with Orthodox Christianity followers. Currently there are five public hospitals in the city. The family planning coverage for 2017 was 51% [16]. The qualitative data was conducted by purposive sampling method to enrich the quantitative data.

A formative evaluation approach was applied because the postpartum contraceptive service delivery was an ongoing process and the findings were anticipated to help and improve the service. The focus of the evaluation was on process of the postpartum contraceptive service delivery involving the inputs, activities, and output components of the service delivery.

### Population and inclusion criteria

All systematically selected charts of reproductive age group women (15–49 years) who gave live or stillbirth during the last 12 months from February 16/2019-February 15/2020 from the selected ACSH maternity ward and from MCH family, four midwives serving in the hospital, 15 interviewed mothers about professionals clinical competence, 10 FP users asked about the compliance of services during exit interview were included in to the study. However, Women who were critically ill after child birth throughout a year, with incomplete data of first year after child birth and having delivery summary from other facility but came for contraceptive to ACSH within 12 months after delivery were excluded from the study.

### Sample size determination and sampling technique

The sample size for quantitative evaluation was calculated by using a single population proportion formula with the assumptions of 95% confidence interval and 5% marginal of error and with the p value = 76% (total compliance of missed opportunity in postpartum family planning in Oromia Omo Nada district health facility) and n = 280 was the required sample size, by adding 10% contingency rate for charts, the final sample size was = 280 + 28 = 308 women’s chart was assessed [17].

**Fig. 1:**
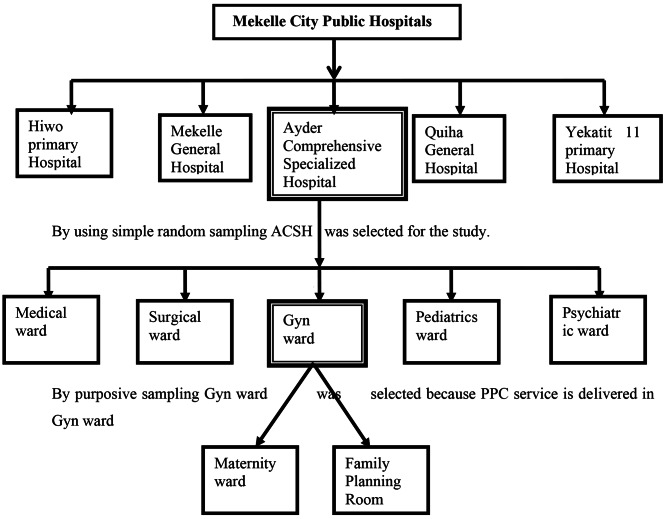
Sampling procedure for PPC service delivery in ACSH

Of the five public hospitals found in Mekelle city, ACSH was selected by systematic random sampling technique. In order to get the sampling interval, the number of delivery registration book of woman which was registered February 16, 2019—February 15, 2020 as source population was divided by the sample sized which yielded 17. After the first women’s medical record number was selected randomly, then every 17th women’s MRN was included in the sample size until the required sample size was achieved. (Fig. 1)

### Data collection tools and procedures

Direct observation checklists, chart review, structured and semi-structured questionnaires were used for data collection. The quantitative part used English version structured questionnaire to review charts of maternal data. In addition, 15(5%) of mothers were observed using an observation checklist to assess the competence of technical and clinical activities of the health care providers.

Direct observation checklist and Key Informant Interview were used to assess the availability of resources and health care professional’s performance and technical competence on FP service. The compliance of services was assessed using direct observation checklist, chart review and exit interview with health care provider. Accommodation of the services was assessed using observation checklist.

The qualitative data used semi structured questionnaire for data collection. Interview guide was used to assess the availability of service with the health care professional and with the mother during exit interview. FP users were asked about the compliance of services during exit interview. Accommodation of the services was assessed using interview with the health care providers. Finally, exit interview was conducted with mothers.

### Data management, quality control and analysis

A pre-test on 5% of the total sample size at similar facility in Mekelle general hospital and amendments were done. One day training was provided for the data collectors before the actual data collection. Daily supervision, on spot checking and reviewing of completed questioners were done. Completed questioners were checked, counted and kept in safe area. Data were cleared and checked manually for its accuracy, missed values and variables. Any logical and consistency errors were identified, and corrected. After data were cleared and checked for its logical consistencies and variables, then, data were interred in to Epi Data version 3.1 and exported to SPSS version 21 software for analysis. Frequency and percentages were done and presented using tables, and figures. In the multivariate logistic regression analysis, variables with < 0.05 *p*-values and Adjusted Odds Ratio (AOR) with 95% Confidence Interval (CI) were used to declare the association. Judgments of the findings of service evaluation were done based on the specific criteria and indicators. Effective program evaluation is a systematic way to improve and account for public health actions by involving procedures that are useful, feasible, ethical, and accurate. The recommended framework was developed to guide public health professionals in using program evaluation [18]. Qualitative data were analyzed manually by coding, categorizing, and them formation.

### Evaluation dimension and judgment criteria

In this evaluation research three evaluation dimensions were used to evaluate the postpartum contraceptive service delivery in ACSH; these dimension were availability, compliance and accommodation.

#### Availability

this dimension was assessed using observation checklist and key informant interview. It assessed the resources such as the adequacy of the supply of contraceptive drugs, essential equipment for the service to deliver, skilled health care professional and other supportive health care providers and the availability of adequate comprehensive postpartum contraceptive service delivery in the facility including infrastructures.

#### Compliance

this dimension was assessed by using chart review and key informant interview with health care providers. It helps whether activities and postpartum contraceptive procedures done to deliver the service in the health facility was done according to the standards of national postpartum contraceptive guideline or not. This dimension determines whether the services provided by the health care providers are compliant to the national guideline standards or not [15].

#### Accommodation

it was tried to look the manners in which the supply resources were organized to accept mother’s needs including appointment system, time of service, and waiting time; keeping privacy and confidentiality, adequacy of toilet and water, adequacy of clean and safe waiting room [19].

### Dimension weights

A nominal technique was used for the allocation of weights among the three dimensions of postpartum contraceptive service with the stakeholder’s agreement based on the degree of relevance. The numbers were given proportionally based on the value judgment related to the importance of the dimensions, areas of the program and indicators to the study questions. Based on this information the dimensions of the availability got 53.14%, of the grand total result, compliance 33.71% and accommodation 13.14%.

The points allocated to each dimensions were divided among the number of indicators and to measure the achievements, assign points per indicators were divided among the acquired points to get percentage score. Each indicators score were aggregated to determine the performance of the program and finally the level of performance of the PPC service were judged based on the final result.

### Matrix of judgment and indicators

This evaluation on PPC service was judged based on the performance of the implementation using the indicators listed below in which the indicators were used as criteria in the judgments of the program. Each indicator, dimension and the overall level of the PPC service operation was judged using the following parameters: 91–100 = Excellent; 81–90 = Very Good; 66–80 = Good; 51–65 = fair; ≤50 = poor. This study used a reference from a similar study done in, Omo Nada district, 2018 based on these parameters 91–100% Excellent, 81–90% Very good, 66–80% Good 51–65% Acceptable26–50% Critical, ≤ 25% No implementation [17].

### Operational definitions

#### Availability

refers to the adequacy of postpartum contraceptive service required resources and infrastructure of the facility.

#### Accommodation

the relationship between the manner in which the supply resources are organized, to accept mothers (including appointment systems, time of service, walk-in facilities, waiting time) and the mothers ability to accommodate to these factors and the mothers perception of their appropriateness.

#### Compliance

it refers to the effort to look for whether activities and procedures are done according to the standards of the national postpartum contraceptive guideline in the study units.

#### Evaluation

is the systematic gathering of in order to make choices among alternative course of action and help improve and upgrade the quality by providing feedback on success and failure.

#### Exit interview

an interview conducted after client has visited the health care provider to check service convenient.

#### Guideline

systematically developed statements to help practitioners and patients make decisions in specific clinical circumstances.

#### Process evaluation

is a type of evaluation which responds for the question (how did or does) and compares the manner in which a program is operating with the plan for the program, and its operation’s oriented.

### Ethics approval

Ethical clearance and approval (ERC1590/2020) was obtained from Mekelle University ethical committee school of public health, department of health system which then submitted to MICHU clinic and maternity ward at ACSH. After explaining the objectives of the study in detail, the participants were ensured of confidentiality of the data. All methods were performed in accordance with the relevant guidelines and regulations. After providing information about the research, informed consent was obtained from all subjects, a parent and/or legal guardian for participants below 18.

## Results

### Socio-Demographic characteristics

A total of 302 (98.5%) mothers’ charts were reviewed. The finding indicated that 217 (71.9%) records belong to Mekelle resident mothers. More than one third of respondents were less than or equal to 24 years of age. About 95% of the respondents were married with the mean age of 27.15 ± 5.46 (SD).

From the data obtained, 256 (84.8%) of them had received at list one history of ANC visit for their recent pregnancy in which 99 (32.8%) of the respondents were their first pregnancy and the rest 46 (15.2%) of them had no documented ANC visit. From total ANC records reviewed, 228 (75.5%) showed that the mothers were counseled about the importance of postpartum contraceptive during their ANC visit. Regarding the mode of delivery, 252 (83.4%) mothers gave birth through spontaneous vaginal delivery (SVD), and 28 (9.3%) was caesarean delivered. Out of the total 302 charts reviewed, 54 (17.9%) records revealed that the mothers had received one or two postnatal care visit (PNC) while the rest 248 (82.1%) of records did not have recorded postnatal care visit.

The time interval between the last two pregnancies was assessed. As a result, 93 (30.8%) of the records indicated that women delivered after two years of their previous pregnancy. Eight (2.6%) indicated that women delivered less than two years of their previous pregnancy which is the main indicator for the unmet need for postpartum contraceptive while the rest 102 (33.8%) of the records were not documented their birth interval **(**Table 1**).**

**Table 1:** Socio-demographic, and obstetric characteristics during PPC service delivery in ACSH

Variables	Frequency	PPC used	
	n (%)	Yes (n,%)	No (n,%)
**Socio-demographic characteristics**			
Age of mothers (in year)			
<=24	105(34.8)	67(35.6)	41(36.0)
25–29	101(33.4)	63(33.5)	37(32.5)
>=30	96(31.8)	58(30.9)	36(31.6)
Residence			
Mekelle	217(71.9)	155(82.4)	62(54.4)
Out of Mekelle	85(28.1)	33(17.6)	52(45.6)
Marital status			
Married	286(94.7)	178(94.7)	108(94.7
Unmarried	16(5.3)	10(5.3)	6(5.3)
**Obstetric history and ANC**			
ANC visits			
1–2	52(17.2)	15(8.0)	37(32.4)
3–4	204(67.5)	137(72.9)	67(58.8)
Not recorded	46(15.2)	36(19.1)	10(8.8)
Gravida			
1	99(32.8)	65(34.6)	34(29.8)
2–4	193(63.9)	120(63.8)	73(64.0)
>=5	10(3.31)	3(1.6)	7(6.1)
Parity			
1–2	108(35.8)	71(37.8)	37(32.5)
3–4	99(32.8)	60(31.9)	39(34.2)
>=5	95(31.5)	57(30.3)	38(33.3)
Mode of delivery of recent delivery			
Normal SVD	252(83.4)	157(83.5)	95(83.3)
Assisted Vaginal delivery	22(7.3)	13(6.9)	9(7.9)
Caesarean delivery	28(9.3)	18(9.6)	10(8.8)
Health care provider who assisted the labor			
Doctor	154(51.0)	96(51.1)	58(50.9)
Midwife	148(49.0)	92(48.9)	56(49.1)
Time Interval between Last Pregnancy			

### Evaluation dimension

The overall evaluation of postpartum contraceptive was 84.1% which indicated a judgment parameter of V/good based on predetermined stakeholder’s matrix of judgment. Whereas, availability dimension was judged as excellent (92%), compliance 71% good and accommodation 85.7% judged as V/good as summarized in the following **(**Table 2**)**.

**Table 2:** The overall evaluation of PPC service delivery in ACSH

No	Dimension	Expected result (%)	Observed Result (%)	No of indicators	Wt	Result	%	Judgment
1	Availability	100	92.0	20.0	53.1	48.9	92.0	Excellent
2	Compliance	100	71.0	15.0	33.7	23.9	71.0	Good
3	Accommodation	100	85.7	6.0	13.1	11.3	85.7	V/good
4	Overall judgment			41.0	100	84.1	84.1	V/good

### Availability dimension

The overall availability of resources in maternity ward and family planning room was judged as excellent (92%). The GP, midwife, laboratory personnel, pharmacy personnel, anesthesia personnel, and gynecologists providing services in the maternity ward and family planning room were one, 50, 96, six, 33, and 14, respectively and judged excellent, very good, very good, very good, excellent and excellent, respectively **(**Table 3**).** From KII, it was revealed that there were three BSc midwives who were trained in basic family planning, one GP and one gynecologist assigned in Gyn OPD for those STI suspected and medically ineligible mothers.

An interviewed 26 years old female midwifery with seven years of work experience from family planning room stated as:

..Since Ayder is a specialized referral hospital, I didn’t see lack of skilled human power. We have adequate staffs; in addition to this, our institution is a teaching hospital that all intern students, all under graduate nurses and midwifery students provide service …

**Table 3:** Results of matrix of judgment and analysis of availability of human resources dimension of the evaluation, ACSH

Evaluation dimension	Wt	Indicators	Values		%	Judgment
			Wt	Achieved		
Determine availability of the needed trained health care providers to run the FP service	56	Gynecology specialist	10	10	100	Excellent
		General practitioner (Dr)	10	10	100	Excellent
		Trained BSc midwife in basic national FP	10	9	90	v/good
		Pharmacy technician	9	8	88	V/good
		Trained laboratory technician	9	8	88	V/Good
		Trained anesthetists	8	8	100	Excellent
		Sub-total	56	53	94.6	Excellent

1. Availability of resources dimension [Weight allocated = 53.14% of the grand total dimension]

1.1 availability of trained health care providers weight allocated 16.% of the total availability dimension

A total of 11 contraceptive methods and 12 necessary materials to run the postpartum contraceptive service delivery as the standard were available. Injectable, implanon, male and female condom, natural method counselling, emergency contraceptive, IUCD, levonorgestrel implants, COC, POP and spermicidal were among checked contraceptive methods. Except the sample contraceptive methods which was judged **very good (88.8)**, all the rest contraceptive methods were judged as **excellent (**Table 4**).** From necessary materials such as Height weight scale, examination table, different kinds of speculum, BP apparatus, stethoscope, disinfectant solutions autoclave machine glove both surgical and clean, uterus sound, sterile gauze, alcohol hand rub and computer data base were judged as **excellent**. Whereas, from the necessary materials there was no female anatomical model which was scored 0 (0%) and judged as poor **(**Table 4**).**

**Table 4:** Availability of modern contraceptive and other necessary materials in ACSH

Evaluation dimension	Wt	Indicators	Values		%	Judgment
			Wt	Achieved		
Determine availability of the needed resources to run the FP service	130	Proportion of the availability of modern contraceptive methods.	10	9.0	90.9	V/Good
		Availability of clinical guideline	9	9.0	100	Excellent
		Availability of national family planning guideline	10	10.0	100	Excellent
		Availability of functional BP apparatus with stethoscope	10	10.0	100	Excellent
		Availability of computer data base	9	9.0	100	Excellent
		Availability of disinfectant solutions	9	9.0	100	Excellent
		Availability of sterilizer machine (autoclave)	10	10.0	100	Excellent
		Availability of glove both surgical and clean	10	10.0	100	Excellent
		Availability of table and chair to receive client for counseling	9	9.0	100	Excellent
		Availability of different kind of posters to guide counseling	9	7.0	77.8	good
		Proportion of the availability of Sample contraceptives	9	8.0	88.8	V/Good
		Availability of functional height-weight scale	9	9.0	100	Excellent
		Availability of clean and safe Examination bed	9	9.0	100	Excellent
		Availability of female Anatomical model	8	0.0	0.0	Poor
		Sub-total	130	118.1	90.8	V/good
		Total availability of resources	186	171.1	92.0	Excellent

1.2 Availability of modern contraceptive methods and other necessary materials weight allocate 37.14% of the total availability dimension

### Compliance dimension

The overall compliance dimension in this study was judged as good (71%). Based on the national guideline, there are clinical assessments and recommendations to provide the contraceptive methods for postpartum mothers before deciding to provide the contraceptive method. It includes assessing STI history, history of contraindication for the method, bad obstetric history, history of TT status, history of chronic illness, check status of HGN and HCT, ask reproductive history of the mother and desire number of children, ask about breast feeding status regularity of menstrual cycle and other more than 30 clinical activities were assessed compared to the national standards.

From the total 302 reviewed charts, 298(98.7%) of the pregnancies were planned, 93(30.8%) of the mothers delivered their second baby > = 25 months, 99(32.8%) were their first birth and the rest 102(33.8%) were not documented (Table 5**).**

**Table 5:** Clinical activities of health care providers before providing family planning in ACSP

Mothers counseled and checked STI and HIV		
	**Frequency (N)**	**(%)**
Test		
Yes	224	74.2
Not documented	78	25.8
Still birth/neonatal loss		
Yes	15	5.0
Not documented	287	95.0
Pregnancy condition of the recent pregnancy		
Planned	298	98.7
Adverse reaction of mother’s after receiving PPC		
Not documented	188	62.3
Unknown	114	37.7
Status of postnatal visit		
Yes	54	17.9
Not documented	248	82.1
Postnatal counseling status of mother		
Yes	36	11.9
Not documented	266	81.1
Maternal counseling during ANC visits		
Yes	228	75.5
Not documented	74	24.5
Total	302	100

From the chart reviewed and observation checklist done, information was provided for mothers by the health care providers but some necessary information was missed. Counseled and checked status of STI and HIV 224 (74.2%) **good**, counseled and checked TT status 281(93%) **excellent**, and history of counseling on family planning during ANC (75.5%) **good**, counseling about possible side effects of the method they receive (100%) **excellent**, appropriate use of the postpartum family planning guideline (70%) **good**, women who receive appointment card (100%) **excellent**, mothers who counseled family planning during PNC **(14%) poor** was measured based on the pre-set stakeholder’s agreed judgment criteria **(**Table 6**).**

For the recent pregnancy, 114(37.7%) of maternal records had completed their fourth ANC visit, 90 (29.8%) complete their third visit, 42 (13.9%) completed their second visit, 10 (3.3%) were with only one visit and the rest 46 (15.2) had not documented ANC visit for their recent pregnancy. After delivery only 54 (17.9%) mothers had received their first or second post natal care visit, and the rest 248 (82.1) mothers had no documented PNC visit and counselling status on postpartum contraceptive service delivery.

From the total of 302 reviewed charts only 188(62.3%) mothers used any method of postpartum contraceptive service after delivery while the rest of 114(37.7%) of the mothers had no document whether they had or not, 82(27.2%) of the mothers used immediately after delivery within 48 h, 28(9.3%) used within 45 days after delivery, and the rest 78(25.8%) of the mothers were used after 45 days of delivery up to one year. Majority of the mothers were used injectable 67 (22.2%) followed by implant 59 (18.5%). Combined oral contraceptive (COC) 1(0.3%), male condom 2(0.7%) and post pills 4(1.3%) were the list prevalent used methods respectively.

Even through clients were well served and program implementation level was (84.1) **very good** according to stakeholders agreed judgments, several technical and skill gaps were observed. It includes asking about sexual history (65%) **fair** mothers who didn’t recorded their birth interval (33.8%) **poor**, mothers who received necessary information before service (87.5%) **very good** proportion of mothers who asked reproductive history **(80%) good**, proportion of women who received respectful activities (85.5%) **very good** was measured based on the pre-set judgment criteria as shown in **(**Table 7**).**

Four of the interviewed health care providers who have work experience of 4–6 years male midwiferies in maternity ward and family planning room stated as:

“….*in our hospital, in addition to giving the contraceptive methods, we check other services such as HIV and STI status, TT status, asking reproductive history of the mother by keeping privacy and confidentiality for service. To ensure this, we close doors and windows before service. In addition, we use privacy box to ensure privacy. Even though our hospital is a teaching hospital, we do not allow more than 2–3 students per case. But, if the mother is not comfortable still with the students, we can provide the service with no students…’’*.

**Table 6:** Judgment and analysis of compliance dimension of PPC with the national guideline in ACSH

Evaluation Dimension	Wt	Indicator	Values			
			*Wt*	*Achieved*	%	Judgment
Compliance with national FP guideline performed activity dimensions	71	Proportion of women who counseled about possible side effect of the method they receive	8	8.0	100	Excellent
		Appropriate use of national FP guideline	8	5.5	70.0	good
		Proportion of women provided PPC after counseling within 12 months	8	5.0	62.2	fair
		Proportion of women who receive appointment card	8	8.0	100	Excellent
		Proportion of PPM who start LARC immediately after delivery within 48 hours	8	2.2	27.1	Poor
		Proportion of PPM who checked TT status during PPC service delivery	8	7.4	93.05	Excellent
		Proportion of mothers who counseled on FP during PNC visit	7	1.0	14.0	poor
		Proportion of mothers who checked STI status during FP service	8	7.5	94.0	Excellent
		Proportion of mothers who counseled on FP during ANC visit	8	6.0	75.5	good
Sub-total			71	50.7	71.4	good

2. Compliance with the FP service to the National FP guideline Implementation Standards [Total weight for the dimension given is 118 points or 33.71% of the grand total weight.

2.1 Compliance with activities from women chart review exit interview and direct observation of activity, weight allocated 20.28% of the compliance-dimension

**Table 7:** Result of health care providers’ technical competence in ACSH

Evaluation dimension	Wt	Indicators	Value		%	Judgment
			Wt	Achieved		
Health care providers technical and clinical competence dimension	47	Proportion of mothers who get respect full activities to use FP service	8	6.8	85.5	V/good
		Proportion of mothers who asked about sexual history before service	8	5.2	65.0	fair
		Proportion of mothers who asked about reproductive history before service	8	6.4	80.0	good
		Proportion of mothers who ask about medical history before service	8	5.2	65.0	Fair
		Proportion of mothers who provided necessary information on FP before service	8	7.0	87.5	v/good
		Proportion of mothers who didn’t record their birth interval	7	2.4	33.8	Poor
		Sub-total	47	33.0	70.3	Good
		Total compliance dimension result	118	83.7	71.0	good

2.2 Health care providers technical and clinical competence dimensions regarding the national FP guideline Weight allocated 40% of the total-dimension

### Accommodation dimensions

The postpartum contraceptive service delivery is functional 24 h a day, seven days a week and 30 days a month in maternity ward for mothers who are volunteers to use the contraceptive methods starting 10 min after placental delivery within 48 h. Therefore this condition makes the service safe and convenient in maternity ward. Whereas, in MCH, the family planning service is delivered eight hours a day and five days a week (Monday to Friday) excluding public holidays, this may make the service inconvenient to use at any time.

During direct observation and an interview made with key informants, there was lack of running water through pipes judged as **good** (75%. This condition indicates that there is scarcity of water for proper implementation of infection prevention in the facility.

From an exit interview made, twelve of the fifteen interviewed mothers reported that the average waiting time to see health care provider was reported 15–30 min which is the recommended average waiting time. Whereas, three of the fifteen interviewed mothers reported that the average waiting time to see health care provider was greater than 30 min up to 60 min measured as proportion of mothers to see health care provider (80%) **good**.

The postpartum contraceptive service in family planning room is standardized, well illuminated, clean and safe, well-kept privacy and confidential with separated counseling room well illuminated room judges as **excellent** (100%). One waiting hall with adequate chairs to seat when mothers are waiting for service, the hall incorporates TV channel as to gain information about maternal health including family planning clean and safe waiting room judges as (87.5%) **very Good.**

Reception room with one chair and table for counseling clients about the available methods and another is for providing the long acting methods and for processing materials service delivery room maintaining privacy of clients (100%) **excellent.** In maternity ward the service is provided after counseling immediate post natal and post caesarean deliveries. There are ten rooms in total for both normal postnatal and post caesarean deliveries with one separated room for the service; the two rooms are for post caesarean delivery, one room is for high risk mothers and the rest six rooms are for normal postnatal. All the post natal and service delivery rooms were clean, safe and well-illuminated during observation. Each room is able to maintain client’s privacy and confidentiality. So the physical conditions of the rooms are perfect in agreement with the standard.

But, even if there are separated rooms for normal postnatal and post caesarean delivered mothers as the recommendation of standards, one health care provider informed as sometimes there is miscible of normal postnatal with caesarean post operation mothers which are high risk for infection due to shortage of rooms and beds.

The interviewed postpartum contraceptive user reports that the waiting room in family planning is convenient for them to serve. Besides, the mother said that the waiting room has a TV channel and adequate chairs to seat. In contrast, another interviewed mother complained that due to inadequacy of water, it is inconvenient to use toilet during waiting time. In general, the accommodation dimension of the PPC service was 85.7% fit to the pre-set criteria and is judged as “**V/Good**” (Table 8**).**

Majority of the interviewed mothers from family planning room about the adequacy of the waiting room, average waiting time and toilet utilization witnessed as:

…*the waiting room is clean, safe with adequate chairs to seat. There was no suffocation during waiting time; with adequate convenience. And, the average waiting time to see health care provider was about 15–30 min*…

To the contrary;

Three of the interviewed mothers from family planning room complained that:

Due to the inadequacy of water, it makes it difficult to use toilet during the waiting time. The average waiting time to see health care provider is more than half an hour or between 30–60 min…

**Table 8:** Results of matrix of judgment and analysis of accommodation dimension of PPC in ACSH

Evaluation dimension	Wt	Indicators	Values		%	Judgment
			Wt	Achieved		
The appropriateness of the physical setup and service delivery (accommodation)	46	Clean and safe toilet for clients	8	6.0	75.0	good
		Clean and safe waiting room	7	6.0	87.5	v/good
		PPC service delivery room maintaining privacy of clients	8	8.0	100	Excellent
		Well illuminated room(s)	7	7.0	100	Excellent
		Source of clean running water	8	6.0	75.0	good
		Proportion of women who get health care provider within half an hour	8	6.4	80.0	good
		Total accommodation result	46	39.4	85.7	v/good
		Grand total of the evaluation	350	294.2	84.1	v/good

3. Accommodation dimension [Total weight for the dimension = 13.14% of the grand total dimension]

### Bivariate and multivariate analysis of postpartum contraceptive

The independent variables such as residence, age, number of stillbirth or neonatal loss, number of ANC visit, counseling status of family planning during ANC visit, counseling status of family planning during PNC visit and gravidity, were found to be significantly associated with proportion of contraceptive adoption on binary logistic regression. However, residence, number of still birth or neonatal loss, counseling status of family planning during ANC visit and maternal counseling status of family planning during postnatal care visits were found to be significantly associated with proportion of postpartum contraceptive adoption on multivariate logistic regression.

Women who were in Mekelle town were 3 times more likely to use postpartum contraceptive compared to their counter parts with [AOR = 3.29 CI(1.76–6.16)] during their postpartum period, similarly mothers who had no still birth or neonatal loss were 8 times more likely to use postpartum contraceptive compared to those who have one neonatal loss with [AOR = 8.29; 95% CI (1.77–38.74)], and mothers who were counselled on family planning during ANC visit were 4 times more likely to use postpartum contraceptive compared to those who didn’t counselled with [AOR = 3.776; 95% CI (1.882–7.575)] during postpartum period and mothers who were counselled on family planning during PNC visit were 2.5 times more likely to use postpartum contraceptive compared to those who were not counseled with [AOR = 2.642; 95% CI (1.38–5.061) during postpartum period **(**Table 9**).**

**Table 9:** Factors associated with PPC use during postpartum period in ACSH Mekelle, Tigray Ethiopia

Variables	Postpartum contraceptive use				
	Yes (%)	No (%)	COR (95% CI)	AOR (95% CI)	P-value
Maternal age in years					
<=24	67(35.6)	39(34.2)	1.00	1.00	
25–29	63(33.5)	39(34.2)	1.34(0.29-1.36)	1.44 (0.64, 3.25)	0.376
>=30	58(30.9)	36(31.6)	1.54(0.19-1.11)	2.09(0.85, 5.12)	0.108
Residence					
Out of Mekelle	33(17.6)	52(45.6)	1.00	1.00	
Within Mekelle	155(82.4)	62(54.4)	3.9(2.32–6.66)	3.29 (1.76, 6.16)	< 0.001
Gravida					
>=5	3 (1.6)	7(6.1)	1.00	1.00	
2–4	120(63.8)	73(64.0)	4.46(1.08–18.35)	0.51, (0.23, 1.14)	0.103
1	65(34.6)	34(29.8)	3.83(0.96-15.29)	0.31, (0.05, 1.83)	0.196
ANC visits					
3–4 visit	135(71.8)	70(61.4)	1.00	1.00	
1–2 visit	16(8.5)	35(30.7)	2.13(0.97-4.66)	0.23, (0.08, 0.67)	0.007
Not recorded	37(19.7)	9(7.9)	0.23(0.12-0.45)	0.64, (0.26, 1.58)	0.333
Neonatal loss or stillbirth					
One loss	3(1.6)	8(7)	1.00	1.00	
No loss	185(98.4)	106(93)	4.65(1.20 17.92)	8.29,(1.77, 38.74)	0.007
Mother counseled on FP during ANC					
No	58(30.9)	16(14)	1.00	1.00	
Yes	130(69.1)	98(86)	2.73(1.48, 5.042)	3.776, (1.88–7.575)	0.003
Mother counseled on FP during PNC					
No	159(84.6)	85(74.6)	1.00	1.00	
Yes	29 (15.4)	29(25.4)	1.87(1.049–3.33)	2.64 (1.38, 5.06)	0.041

NB, COR = crude odd ratio, AOR = adjusted odd ratio, significant statistically at P-value < 0.05

## Discussion

This process evaluation assessed PPC service delivery using three dimensions, postpartum contraceptive service facility compliance to the national family planning implementation standards (focusing on availability, compliance and accommodation dimensions), health care providers’ technical compliance to the national guideline and general settings of the postpartum contraceptive service.

Our study pointed out the availability of 11 types of modern contraceptive methods obtained using availability checklist. As a result, almost all modern contraceptive methods except spermicidal were checked during observation and the evaluation result when compared with the predetermined criteria and parameter was **V/good** (90.9%). This study is relatively higher than a facility based evaluation done in Oromia region which had four of the six assessed contraceptive methods that yields (66.6%), and scored as **good** [17].

Concerning the availability of necessary materials to run the service, it was comparable to the national family planning guideline. It includes the availability and functionality of 12 essential materials to run the service in each study unit; all the necessary materials were comparable with the national family planning standards except the female anatomical model. However, materials in store or material not properly functional during observation were not available for the service [14].

In general settings of infrastructure the family planning service in MICHU clinic has three separated rooms. One waiting room with adequate chairs to seat for mothers, one reception room to receive and clerk mothers and one separated room for counseling and physical examination and to provide for long acting methods. Accordingly, all those rooms are well illuminated, clean and safe, well-kept privacy and confidential which is comparable to the national standard [14].

The compliance of the PPC service of the facility was judged **good** (71%) compared to the national postpartum family planning implementation standards according to the pre-set judgment criteria and parameter. Accordingly, during the postpartum period, only 27.2% of the postpartum mothers used the long acting reversible contraceptive (LARC) during the immediate postpartum up to 48 h. Evidence from Kenya indicated that by nine months after child birth, 59% of women used modern contraception, however, only 12% used long acting reversible contraceptive (LARC) methods [8].

This evaluation result reported that 8(2.6%) mothers gave birth of their second pregnancy less than 24 months, similarly analysis of DHS data in 60 USAID assisted middle income countries found that 22.5 million adolescents age of 15–19 gave birth, from the 22.5 million total adolescents approximately 4.1 million adolescents gave birth to their second pregnancy within 24 months due to lack access to postpartum contraceptive [20].

The extended postpartum methods given after 45 days of delivery were 78 (25.8%). A study conducted in North West Ethiopia on extended postpartum indicates that a total of 10.3% of the mothers reported adopting contraception in the extended postpartum period [21]. However, the extended postpartum conducted in North West Ethiopia was started after six months of postpartum period, where as in this study the extended postpartum was measured 45 days after delivery. Likewise, in West Ethiopia, modern family planning in extended postpartum period was 45.4% which was highly influenced by antenatal post-natal care visits [22].

In this evaluation, the total prevalence of postpartum contraceptive utilization found to be62.3% which is higher than the study done in Tigray region, with the overall contraceptive prevalence rate among all women was 623 (35.6%) while 543 (41.0%) among married women [12], 38.3% in rural setting of Tigray [13], 38.9%in Amhara Regional State Zonal Towns, North West Ethiopia [23]. However, this study is lower than a study conducted in Addis Ababa 80.3% [24], Kenya (86.3%) [25], and Uganda (28%), used modern contraceptive method [26]. The differences could be due to socio-economic, service quality and study area differences across study settings.

Women who reside in Mekelle town were 3 times more likely to use postpartum contraceptive compared to those who live out of Mekelle town during their postpartum period. This is consistent with a study done in north Ethiopia Tigray that the odds of using family planning by married women living in urban area was 2 times more than those who reside in rural areas [12].

Finding of this evaluation showed that the use of postpartum contraceptive during postpartum period has significantly associated with maternal health care service. Women who had ANC visit during pregnancy were more likely to use postpartum contraceptive during postpartum period which shown similarity with a study done on postpartum family planning [27].

Women who had PNC visits were more likely to use postpartum contraceptive during postpartum period. Similar study done in southern Ethiopia, in Butajira on postpartum contraceptive adoption within 12 month supported this study [28]. This could be due to the fact that women who get information during ANC and PNC from health professionals about the family planning methods might have improved the likelihood of using PPC. Getting information about family planning before delivery facilitates decision for adopting the method very early after delivery. Another study done in Northern Ethiopia about postpartum modern contraceptive use shows that family planning counseling during pre-natal care and postnatal care were positively associated with postpartum contraceptive use during postpartum period [29].

Coordination of the health system such as integration of service and providing linkages between ANC, birth service, PNC, child health and family planning service may insure continuity of care and access to postpartum contraceptive [14]. This study also tried to assess service integration during postpartum contraceptive such as checking TT status, STI history, blood group and hemoglobin. Thus, this study fitted to the national standard. Whereas, family planning counseling during ANC and PNC visits was measured as poor based on the pre- set stakeholder’s agreed analysis and judgment which requires some improvement to fit with the national standard.

In this evaluation research shows that the long acting reversible contraceptive was found to be lower than the short acting, in which injectable accounts 67(22.2%), implanon accounts 59 (18.5) and IUCD accounts 20(6.6%). Similarly, a recent evidence from Adigrat indicated that the post-partum long acting reversible contraceptive(LARC)service utilization was as low as 5.8% (comprised of 5.3% Implants and 0.5% Intrauterine device (IUD), while the short acting family planning service coverage was as high as 94.2% [6]. In this evaluation research, injectable was the more prevalent method followed by implanon 22.2% and 18.5% respectively, similarly evidence from the rural setting of Tigray indicated that 33.8% of postpartum mothers used modern contraceptives. Depot medroxyprogesterone acetate and implant were the most prevalent methods used [12].

This study revealed that all women had received their appointment card and were told where and when to go for the next supply by the health care provider which is performed according to the recommended the national standard. This study shows similar finding with a study done in Oromia in Omo Nada district and was measured 100% performed as the national standards [17]. In addition, mothers were informed to come before the appointment if the mother feels discomfort with the method she received.

In case of measuring clinical competency of health care providers, the result of this study revealed that, even though clients were well served and program implementation level was very good according to the stakeholders agreed judgment, some clinical activities were missed during observation checklist and chart review. Some of the missed clinical activities were, poor information provision, poor technical competence, ignored for oral consent for the procedure, poor emotional support, and failure to proper procedure for infection prevention procedures activities. In Ghana, about 6.0% and 4.5% were dissatisfied about auditory and visual privacy during counselling respectively. Most of the clients (79.1%) were not given educational materials although 88.8% were talked to about family planning [30].

As discussed in the result part, PPC in maternity ward is available 24 h a day. Therefore, if the mother is willing to use the method at any time, this condition makes the service safe and convenient at any time. So as to improve unmet need of family planning, as the national guideline recommends that the service should be accessible at any time [15]. Another study conducted in Malawi revealed that almost two-thirds of faith-based facilities were open five or more days/week, which is less than other private facilities (85%) but compares favorably to the public sector (49%) [31].

Regarding the convenience of postpartum contraceptive in terms of source of water, even though there is a pipe system of water, there is limitation of water as confirmed with the observation checklist and the witness of the study participants. They didn’t satisfy with the available amount of water. Unlikely, a study done in Oromia region in Omonada district reported that water was the most available resource [17].

### Strength and limitations

We assessed quality using different components such as, observation checklist, key informant interview and facility audit to ascertain availability of resources (methods, providers and the necessary equipment) than using a single dimension. We employed a case study using mixed methods study. However, covid-19 was a challenge to conduct an interview with mothers and key informants, some health care providers who didn’t take the basic training for PPFP caused difficulty to judge them per the national standard, difficulty to limit the number of staffs to ensure the privacy of mothers and work load were some of the limitations encountered.

### Conclusion

The overall level of PPC was found to be V/Good. Compliance of the facility in fulfilling a healthy operation of the service was also Good (71%). In compliance dimension, however, poor infection prevention practice, lack in service trainings, poor use of clinical checklist during counseling, ignored clients to introduce self and position during service and missing vital information were among the identified clinical and technical skill gaps. The availability dimension, the general set up and accommodation of PPC service was measured as excellent and very good with significant gaps that require improvement.

Finally, residence, number of stillbirth or neonatal loss, counseling status of family planning during ANC visit, and maternal counseling status of family planning during postnatal care visit were factors associated with PPC.

### Recommendations

#### The Tigray regional health bureau

Health Extension Workers and other community based professionals should have a built system to improve PPC with a strong supportive supervision. Knowledge and skill gap of health care providers should be updated by providing training on family planning and other basic trainings.

#### ACSH

Should respond to the scarcity of water in the hospital. Maternity ward should have a separate laboratory service to facilitate emergency cases. The hospital has to prepare additional beds and rooms to solve the mixing of normal postnatal with post caesarean mothers which is high risk to infection. Additional payment for night duty and public holidays may be a means to motivate their financial needs.

#### To health care providers

Strictly follow the national guideline standards for counseling and providing the PPC, fill all points in the registration book of family planning. Use clinical checklist during counseling of contraceptive methods in order to avoid missing of essential ideas during counseling. Give due attention in providing adequate time for counseling before providing postpartum contraceptive to improve quality service and to achieve the national standards. During counseling, providers should not promote (over emphasize) on specific family planning methods so that clients make decisions based on their choice of methods. Health care providers should emphasis and strengthen the linkage of women to family planning services when postpartum women come for immunization service.

## Data Availability

The datasets analyzed during the current study are all included within the manuscript itself.

## Declarations

### Disclaimer

None declared.

## Abbreviations

ACSH: Ayder Comprehensive Specialized Hospital
CR: Chart Review
DHS: Demographic and Health Survey
EA: Evaluability Assessment
FMOH: Federal Ministry of Health
FP: Family Planning
HID: Health Information Dissemination
HM: Hospital Managers
HMIS: Health Management Information System
IEC: Information Education Communication
KAP: Knowledge Attitude and Practice
LARC: Long Acting Reversible Contraceptive
ME: Monitoring and Evaluation
MCH: Maternal and child Health
MCHO: Mekelle City Health Office Maternal Mortality
NGOs: Non-Governmental Organizations
NM: Neonatal Mortality
PPC: Postpartum Contraceptive
PPFP: Postpartum Family Planning
TRHB: Tigray Regional Health Bureau
WHO: World Health Organization
